# Insight into Oxygen Transport in Proton Exchange Membrane Water Electrolyzers by In Situ X‐Ray Characterization

**DOI:** 10.1002/advs.202405658

**Published:** 2024-09-26

**Authors:** Ping'an Li, Zihan Zhou, Diankai Qiu, Linfa Peng

**Affiliations:** ^1^ State Key Laboratory of Mechanical System and Vibration Shanghai Jiao Tong University Shanghai 200240 P. R. China; ^2^ Shanghai Key Laboratory of Digital Manufacture for Thin‐walled Structures Shanghai Jiao Tong University Shanghai 200240 P. R. China

**Keywords:** critical current density, in situ, oxygen transport, PEMWE, X‐ray

## Abstract

The proton exchange membrane water electrolyzer (PEMWE) is one of the most promising electrochemical energy conversion devices for hydrogen production, while still limited by performance bottlenecks at high current densities, due to the lack of mass transfer insights. To investigate the mechanisms of oxygen transport inside the PEMWE at high current density and its relation to electrolytic performance. Operational in situ x‐ray imaging is utilized to simultaneously characterize the bubble behavior and voltage response in a novel designed visual mini‐cell, and it is identified that oxygen evolution and transport in the PEMWE follow the process of bubble nucleation, growth, and detachment. Based on the results of mini‐cells with three porous transport layers (PTLs) up to 9 A cm^−2^ operation, it revealed that critical current densities exist for both carbon‐based and titanium‐based PTLs. Once exceeding the critical current density, the cell voltage can no longer be stabilized and the cell exhibits a significant oxygen overpotential. To illustrate this, the concept of interfacial separation zone (ISZ) is first proposed, which is an effective pathway for bubble growth and separation and the pattern of the ISZ exhibits specific regimes with the critical current density. Ultimately, a new approach for better understanding the mechanisms of oxygen transport is revealed.

## Graphical abstract

The oxygen bubble dynamics inside the PEMWE from 0 to 9 A cm^−2^ is in situ revealed by a novel visual mini‐cell. Oxygen transport in PTL still follows the process of bubble nucleation, growth, and detachment. It is found that the interfacial separation zone (ISZ) is an effective pathway for bubble transport and the pattern of the ISZ exhibits specific regimes with the critical current density.

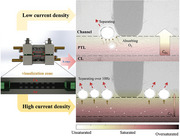

## 1 Introduction

Hydrogen, a high‐specific energy, environmentally friendly fuel, is hailed as the most promising clean energy source of the 21st century. For hydrogen production, a proton exchange membrane water electrolyzer (PEMWE), splits water into hydrogen and oxygen by electricity, which is characterized by high efficiency, fast dynamic response and high‐pressure gas collection. Especially when coupled with renewable energy sources, hydrogen produced by PEMWE is called “green hydrogen” and can be truly zero carbon emission. It is also very attractive in numerous application prospects, due to the high purity hydrogen produced by PEMWE can be directly used in fuel cells, carbon neutralization, and electro‐hydrogen conversion systems.

However, the commercial spread of PEMWE is still limited by its high capital expenditures, primarily owing to titanium and precious metal catalyst materials. Therefore, a strategic means for reducing the capital expenditure of PEMWE is operating at high current densities, thereby reducing the number of cells required at the same power level.^[^ ^1^, ^2^ ^]^ Operating expenses also need to be maintained. It implies that cell performance needs to be further improved to prevent excessive voltage. Currently, PEMWEs are currently operated over the 1–3 A cm^−2^ range and state‐of‐the‐art technology can be operated at 3–5 A cm^−2^. While the optimal current density is expected to increase up to 10 A cm^−2^ to reduce expenditure in the near future.^[^ ^3^ ^]^

While high current density is always accompanied by higher overpotentials leading to lower electrolysis efficiency. This is partly due to the linear increase in ohmic overpotential with electrical density. Moreover, it cannot be ignored that large amounts of oxygen are inevitably produced by the oxygen evolution reaction (OER) at the anode side, and complex water‐gas transport will occur in the porous transport layers (PTLs)^[^ ^4^, ^5^ ^]^ of the anode side. Excessive oxygen evolution and transport can cause severe bubble issues. In recent reports,^[^ ^6^, ^7^ ^]^ bubble issues have been summarized as the high bubble coverage, slow bubble detachment and gas accumulation in PEMWE, while the negative performance impacts caused by bubbles are still difficult to explain clearly which are only attributed to activation loss, ohmic loss, or mass transport loss. This is because most of the descriptions of bubbles in PTL are based on theoretical extrapolations and simulations, whereas the evolution and transport processes of bubbles occurring inside real cell structures are currently still understudied.

In fact, it has been very difficult to precisely measure the behaviors of bubbles inside PEMWE at the micro‐scale. Most previous studies of bubble behaviors have been carried out through mathematical models or in ex situ boiling systems,^[^ ^8^, ^9^, ^10^ ^]^ while a few studies have investigated bubble issues in situ using imaging techniques, including optical, neutron, or X‐ray imaging. Mo et al.,^[^ ^11^ ^]^ utilized a high‐speed and optical micro‐scale visualization system to reveal oxygen bubble dynamics in a transparent cell. It was concluded oxygen evolution only occurs in the form of bubbles along the catalyst layer（CL)/PTL interfaces at the rim of pores on thin titanium PTL and the separation diameter of oxygen bubbles increases with the operating current density. However, optical methods are intrinsically incapable of observing the PTL interior and oxygen transport behavior in the through‐plane direction. Neutron‐based imaging has been utilized by Panchenko et al.,^[^ ^12^ ^]^ to observe oxygen transport processes. It was possible to visualize the water‐oxygen distribution in the cell during operation. Lee et al.,^[^ ^1^ ^]^ also employ operational neutron imaging to quantify the gas saturation inside the PEMWE, and it was concluded the bubble growth at the through‐pore is faster. However, the low resolution and long sampling intervals of the neutron method make it difficult to instantaneously characterize the dynamic behaviors of oxygen on a micro‐scale. Operando X‐ray imaging has been utilized by Leonard et al.,^[^ ^13^ ^]^ for a series of sub‐second radiography studies. Steady‐state water distribution and dynamic oxygen evolution were observed by combining X‐ray computed tomography (CT) and radiography, and the evolution of oxygen bubbles was found to be related to current density. In their following studies,^[^ ^14^ ^]^ proposed oxygen distribution in the PTL had a periodic behavior, and oxygen transport has preferential pathways under current densities (1–4 A cm^−2^). However, due to low material lining, these findings were made possible by combining machine learning techniques to compensate for the lack of X‐ray imaging quality. Additionally, it has also failed to achieve higher current density observations.

It can be seen that there is no consensus among existing studies on the evolution and transport processes of oxygen inside PEMWE. It is still unclear about the bubble's transport behavior and their impacts at high current density, and whether the transmission follows preferential pathways. Therefore, further in situ characterization of PEMWE at high current densities is urgently needed.

This work aims to investigate the mechanisms of oxygen removal inside the PEMWE at high current density and its relation to electrolytic performance. Operational in situ X‐ray imaging is utilized to simultaneously characterize the bubble behavior and voltage response of visual cells from cross‐section views. Clear and high‐speed imaging is vital. Hence, in the second section: the visual cell design and imaging platform solutions will be highlighted to compensate for the limitations of X‐ray for water and gas imaging. In the third section: Based on image frame processing, the bubbles evolution and transport behavior at different current densities and the cell voltage response will be analyzed. In the fourth section: the relationship between the bubble separation zone of PTL on cell performance is discussed through the quantitative statistics of the bubble. The effect of PTL hydrophobicity is also considered. Ultimately, The relationship between the bubble removal mechanism on PEMWE performance at high current density is revealed for the first time.

## 2 Experimental Section

### 2.1 In Situ X‐ray Experimental Platform

Operando X‐ray imaging was conducted at the beamline (BL13W1) at the Shanghai Synchrotron Radiation Facility (SSRF). The photon energy range of the beamline is 8–72.5 keV, Several different pixel sizes (0.19–24 µm) were available to realize phase‐contrast imaging with high resolution.^[^ ^15^ ^]^ To enhance the image contrast between water and gas, the optimized configuration of the beamline was as follows: The X‐ray energy was selected at 13 keV, coupling 2x lenses and 10x lenses, The resulting images had an effective pixel size of 0.325 µm and the field of view was 2.5 mm × 2.5 mm. The temporal sampling rate was 10 Hz (10 frames/s) for the best signal‐to‐noise ratio.

### 2.2 Visual Mini‐Cell Design

**Figure 1:**
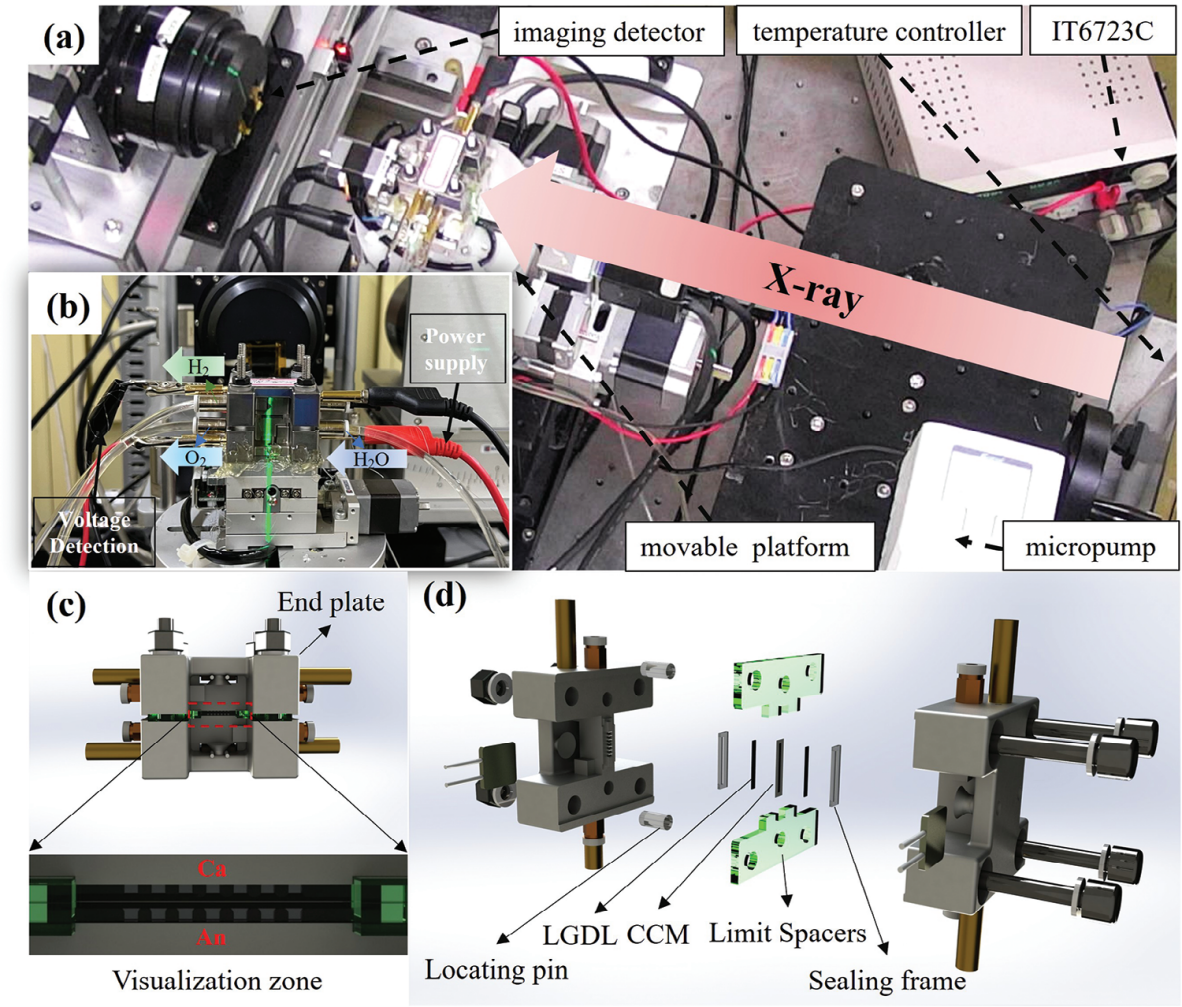
a) Operational in situ X‐ray imaging system from a top view. b) Operational visual mini‐cell from a front view. c) visualization zone in mini‐cell. d) Structural design of the mini‐cell.

To achieve the observation of the mini‐cell in a through‐plane direction, the visualization zone should be designed to penetrate the front view of the cell. As shown in **Figure** **1**b–d, the mini‐cell consists of two end plates, and a visualization zone, which was eight by four bolts. The end plates were milled from titanium alloy to form parallel flow fields with 0.5 mm channel widths. The visualization zone dimensions were 12 mm × 0.9 mm × 0.6 mm (W × D × H) with an active area of 0.09 cm^2^. It consists of a catalyst‐coated membrane (CCM), PTLs, limit spacers, locating pins, and a sealing frame, which was controlled by 20% compression of PTL by displacement. The CCM was provided by Wuhan WUT New^[^ ^16^ ^]^ using membrane Nafion 115 with 2 mg cm^−2^ IrO_2_ in the anode and 0.8 mg cm^−2^ Pt in the cathode. To enhance X‐ray transmission,^[^ ^17^ ^]^ carbon fiber material with low density was preferred as PTL, and the width of PTL was limited to 0.9 mm. In addition, the sealing frames were also made of a silicone material with high transparency.

**Figure 2:**
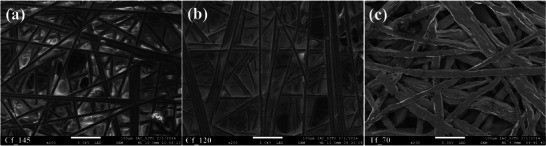
SEM images of different PTLs from top‐view a) Carbon fiber with 145° contact angle. b) Carbon fiber with 120° contact angle. c) Titanium felt with a 70° contact angle.

To illustrate the applicability of this study for commercial PEMWEs, in the comparative experiments, carbon fiber and titanium felt materials were selected as PTLs respectively. The three PTLs are shown in **Figure** **2**, to investigate the effect of different hydrophobicity, two carbon fiber materials were treated by immersion with 5 and 70 wt.% concentrations concentrations of Polytetrafluoro‐Ethylene (PTFE) emulsion respectively, while the titanium felt was left untreated. Previous studies^[^ ^18^, ^19^, ^20^ ^]^ have proved that PTL based on carbon or titanium materials does not cause severe corrosion within 2 h of operation. Finally, three samples were obtained with contact angles of 145°, 120°, and 70°, and more details are shown in **Table** **1**.

**Table 1:** Detailed parameters of the three PTL samples.

sample	Thickness	Width	Porosity	Bulk density	contact angle	PTFE treatment
Carbon fibre (Cf_145)_	0.28 mm	0.9 mm	78%	0.32 g cm^−3^	145°	70 wt.%
Carbon fibre (Cf_120)	0.28 mm	0.9 mm	78%	0.32 g cm^−3^	120°	5 wt.%
Titanium felt (Tf_70)	0.28 mm	0.9 mm	78%	/	70°	/

The three samples exhibit the same porosity and volume. Carbon fiber with a density of 0.32 g cm^−3^ leads to higher X‐ray transmission, while titanium felt will lead to worse imaging quality due to much higher bulk density.

### 2.3 Measurement Protocol

As shown in Figure 1a, the mini‐cell was placed on a movable platform for the in situ X‐ray observation. The in situ platform could be automatically controlled remotely and it consists of a programmable power supply (IT6723C, ITECH), micropump, temperature‐controlled water container, and imaging detector. The micropump was set to 18 mL min^−1^ to ensure an adequate water supply. The mini‐cell was controlled at a constant 65° by the temperature control modules.

To better observe the two‐phase behavior and voltage response under different current densities, the measurement protocol was divided into three parts: In the initialization stage, the dry state of the cell without a water supply was first photographed, after the water supply, until the cell was filled with deionized water and the temperature reaches 65 °C, the oxygen‐free state was photographed as a comparison. In the constant‐density operation stage, the cell was sequentially operated at 12 different current densities in the range of 0–9 A cm^−2^ for 6 min at each current point, and the visualization zone was continuously imaged. In the polarization stage, the polarization curve was measured from 9 to 0 A cm^−2^ for load shedding data, and run for 3 s at 0.01 A cm^−2^ intervals to evaluate the electrolysis performance with the oxygen transport behavior.

## 3 Result

### 3.1 Cell Performance

**Figure 3:**
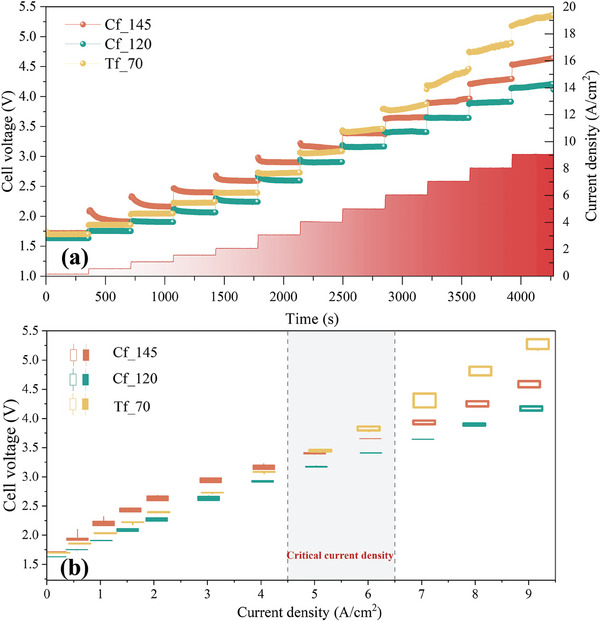
a) In situ voltage response of three mini‐cells under constant densities in the range of 0–9 A cm^−2^ with its corresponding b) candlestick charts (When the starting voltage is higher than the terminal voltage under constant electric density is defined as a negative line, indicated by solid, and conversely, it is defined as positive line, indicated by a hollow. The critical current density is in the interval where the negative line transforms into a positive line).

The voltage responses of the three samples show a common trend as shown in **Figure** **3**a. As the constant current density point increases, the cell voltage increases near‐linearly. However, there are distinctly different increase patterns in the low, medium, and high current density intervals. When the current density is less than 4 A cm^−2^, the voltage shows a fluctuating downward to steady tendency in constant‐density operation. It can be attributed to more reaction sites being progressively activated with the reaction;

When the current density is between 4 and 6 A cm^−2^, the voltage response remains relatively steady within 10 mV of voltage fluctuation; While when the current density is higher than 6 A cm^−2^, the voltage response is no longer smooth and shows a tendency to rise sharply. Such a voltage response pattern will be easier to distinguish after transforming it into a candlestick chart as shown in Figure 3b. As the current density increases, the transition from negative to positive lines is observed for all three samples. Based on this, we define the current density when the voltage response shifts from a smooth to a sharp increase as the critical current density,^[^ ^21^ ^]^ which indicates that the cell is no longer able to operate stably beyond the critical electrical density. It will also be possible to evaluate the cell performance by the critical electric density. It should be noted that the critical current densities are all revealed above 4A/cm^2^ with slight variations between titanium‐based and carbon‐based samples. The phenomenon of sharp overpotential growth, once the critical current density is exceeded, will be further investigated from the perspective of in situ observations.

### 3.2 In Situ Characterization

**Figure 4:**
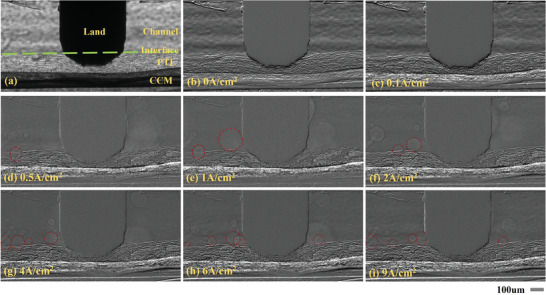
The cell cross‐sectional radiographs of Cf_145 sample a) in the initialization stage with full water and in the constant‐density operation stage for different current densities of b) 0 A cm^−2^, c) 0.1 A cm^−2^, d) 0.5 A cm^−2^, e) 1 A cm^−2^, f) 2 A cm^−2^, g) 4 A cm^−2^, h) 6 A cm^−2^ and i) 9 A cm^−2^ (interfacial separation zones indicated by red lines). S2

Images captured after initialization in the minicell measurement protocol can be used for oxygen behavior studies. Better imaging results were obtained for the Cf_145 sample, as shown in **Figure** **4**. One can observe channels, lands, PTLs, and catalysts on the anode sides of CCM in the same slice. The channel can be observed with a width of 500 um on both sides of the ridge, and the PTL under the land is more compressed than under the channel.

It is not obvious to distinguish the water phase and oxygen phase, due to they have comparable X‐ray attenuation. Therefore, all captured images need to be divided with the full water state of the mini‐cell (Figure 4a), and image processing by bandpass filtering should be performed to optimal contrast. As in Figure 4b‐i, the oxygen phase can be better recognized in the interface region between the channels and the PTLs.

From the cross‐sectional radiographs under a variety of current densities, Oxygen is incrementally distributed as bubbles from the PTL to the channel. The distribution pattern in the flow channel is difficult to determine. This is because there is always an interference of oxygen carried from upstream in the flow channel. However, in the interface region between the PTL and the flow channel, different forms of gas clusters can be clearly recognized as the current density increases. This is because the interface is a critical pathway for bubble convergence and separation. Therefore, it is essential to focus on the bubble behavior in the interface region which is considered to have a critical impact on the cell performance.^[^ ^22^, ^23^, ^24^, ^25^ ^]^ And specific oxygen bubble behavioral regimes can be summarized at a wide variety of current densities

**Figure 5:**
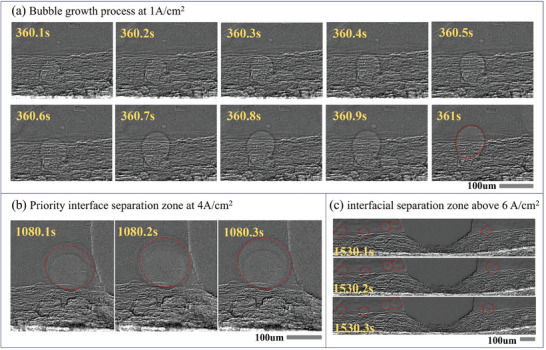
The cell cross‐sectional radiographs from continuous image stream for a) 1 A cm^−2^, b) 4 A cm^−2^, and c) 6 A cm^−2^ current density. (interfacial separation zones indicated by red lines). S1

Starting from 0.5 A cm^−2^ current density, growing bubbles near the interfacial region (Figure 4d) and bubbles adhering to the interfacial region (Figure 4e) are observed. In fact, bubble growth dynamics can be observed in the image stream with a sampling frequency of 10 Hz, as shown in **Figure** **5**a. The bubble formation starts with nucleation,^[^ ^26^, ^27^, ^28^ ^]^ and the bubbles start to nucleate at the bottom of the PTL adjacent to the catalytic layer. This is due to the fact that oxygen is generated at the three‐phase reaction (TPR)^[^ ^29^ ^]^ site at the interface between the catalyst layer and the PTL and is more likely to reach a supersaturated concentration adjacent to the catalyst layer.^[^ ^30^ ^]^ As the oxygen bubble grows, the bubble gradually reaches the interface between the channel and the PTL. This entire process can take upward of 1 s until the dynamic balance of the bubble is broken under a larger dragging force and it escapes from the interface region.

When the current density increases from 1 to 4 A cm^−2^ (Figure 4f,g), more bubbles are observed in the interfacial region growing to separation. It is worth noting that bubbles in certain interfacial regions preferentially stabilize as the current density increases. This is because the produced oxygen is exhausted to the flow channel with a higher frequency. Similar studies have shown that at higher current densities, the separation frequency of oxygen bubbles will approach 10 Hz.^[^ ^13^, ^31^ ^]^

We define every individual region formed by the detachment of oxygen bubbles near the interface from each image frame at a sampling frequency under 10 Hz as the interfacial separation zone (ISZ). Actually, oxygen is preferentially exhausted through the ISZ. Moreover, as the rate of oxygen generation increases at higher current densities, more ISZs are observed to stabilize, which indicates that bubbles are detached from the ISZs at a high frequency close to 10 Hz. To reveal this, from Figure 5b, it can be observed that new gas clusters with different densities are produced with an interval of 0.1 s inside the ISZ. This implies that inside the ISZ, there are new gas clusters that are generated and separated at a frequency close to 10 Hz.

Once the current density exceeds 6 A cm^−2^ (Figure 4h,i), the observable oxygen exhaust behavior seems to shift to a steady state, at which point the dynamic growth of bubbles can barely observed. Moreover, the position of the ISZ has stabilized and the number of it also reaches an upper limit. As shown in Figure 5c for this stage, due to the separation frequency of the oxygen bubbles being higher than 10 fps, which is completed within 0.1 s, the ISZs appear to be stationary from the image stream. This is correspondingly related to the deterioration of cell performance in Section 3.1, in which mass transfer polarization^[^ ^32^ ^]^ arises at the critical current density. One plausible explanation is that oxygen transport has been somewhat impeded and it is difficult to generate new ISZs in the limited interfacial space to promote oxygen bubble separation. It will be discussed in detail in Section 4.

In short, the oxygen transport behavior can be summarized by Figure 5 in three stages with different characteristics:S2 When the current density is much less than the critical electric density, intermittent exhaustion of oxygen bubbles can be observed, accompanied by a complete process of nucleation, growth, and detachment.^[^ ^33^ ^]^ When the current increases to the critical current density, the bubble separation frequency also increases, and more ISZs can be observed sequentially. Once the critical current density is exceeded, the observable ISZs seem to stabilize, and no more ISZ occurrences can be observed. It is worth noting that the above three oxygen transport behaviors strongly correspond to the different stages of cell performance, and that the ISZ is also accompanied by the emergence of the critical current density. Therefore, further quantitative analysis is worthwhile to clarify the intrinsic mechanisms of oxygen transport.

## 4 Discussion

The in situ results above have revealed that oxygen transport behavior at high current densities is still exhausted in the form of bubbles through the ISZs. Actually, the ISZs are contours formed by the residual shadow of the separated bubbles, reflecting characteristics such as the number of bubbles and the size of the separation. In addition, since the oxygen separation behavior between the flow channel and the PTL is the key to the electrolysis performance, it is worthwhile to further numerically analyze the bubbles in the ISZ.

### 4.1 Statistical Analysis of ISZs

Statistical analysis of ISZs requires frame‐by‐frame identification for the cross‐sectional radiographs from image streams.S1 Here, we sample the image streams within 3.2 s for each current density in the range of 1–9 A cm^−2^ from the constant‐density operation stage.

**Figure 6:**
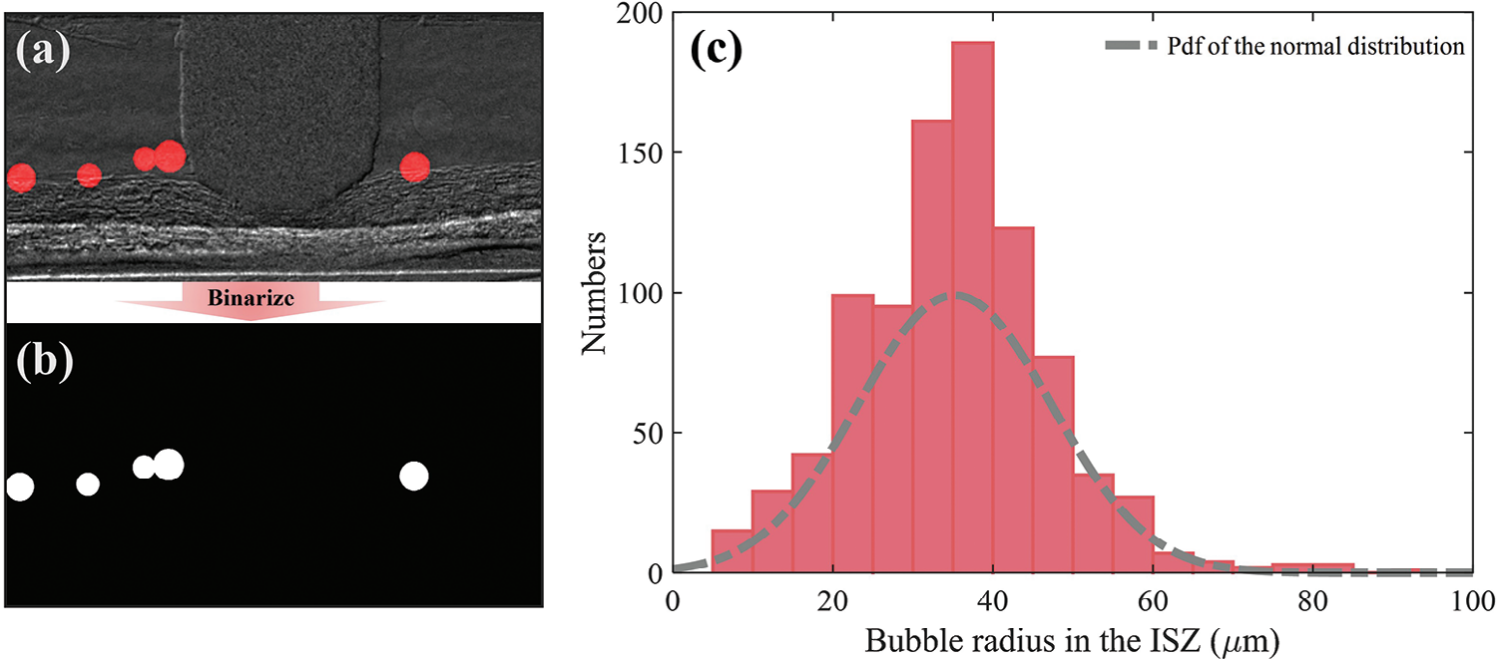
a) Samples labeled with ISZs from image streams and b) binarized samples for quantitative analysis. c) The radius follows the normal distribution probability density function.

As shown in **Figure** **6**a,b, the ISZ of each image is marked with bubbles. To improve statistical accuracy, The bubbles marking process is based on the “Labelme” package in Python 3.11, and anomalous bubbles suspended in the flow channel are excluded from marking. For quantitative analysis, the Image binarization process is performed in Matlab2022a. Then, the embedded function “bwconncomp”^[^ ^34^ ^]^ in Matlab is used to compute the pixel connectivity of the binary image, which in turn counts the position, number, and size of the ISZs. As shown in Figure 6c, a total of 352 images were analyzed for the interfacial separation zone, and more than 900 ISZs were counted. bubble radius of the ISZs ranged from 10 to 90 µm, while their radiuses followed a normal distribution with a mean of 35.3 µm and a standard deviation of 12.1 µm. More than half of the ISZs have radius between 30 and 45 µm, and the other most of the larger or smaller radius are counted in the statistics when the bubbles are in the growth phase at low current densities. This is evident in our counting process, where a change in the growth of the radius of the bubble is observed when below the critical current density, while the radius of the ISZ remains essentially unchanged when above the critical current density. This is due to the bubble separation frequency above the critical current density being close to the sampling frequency.

**Figure 7:**
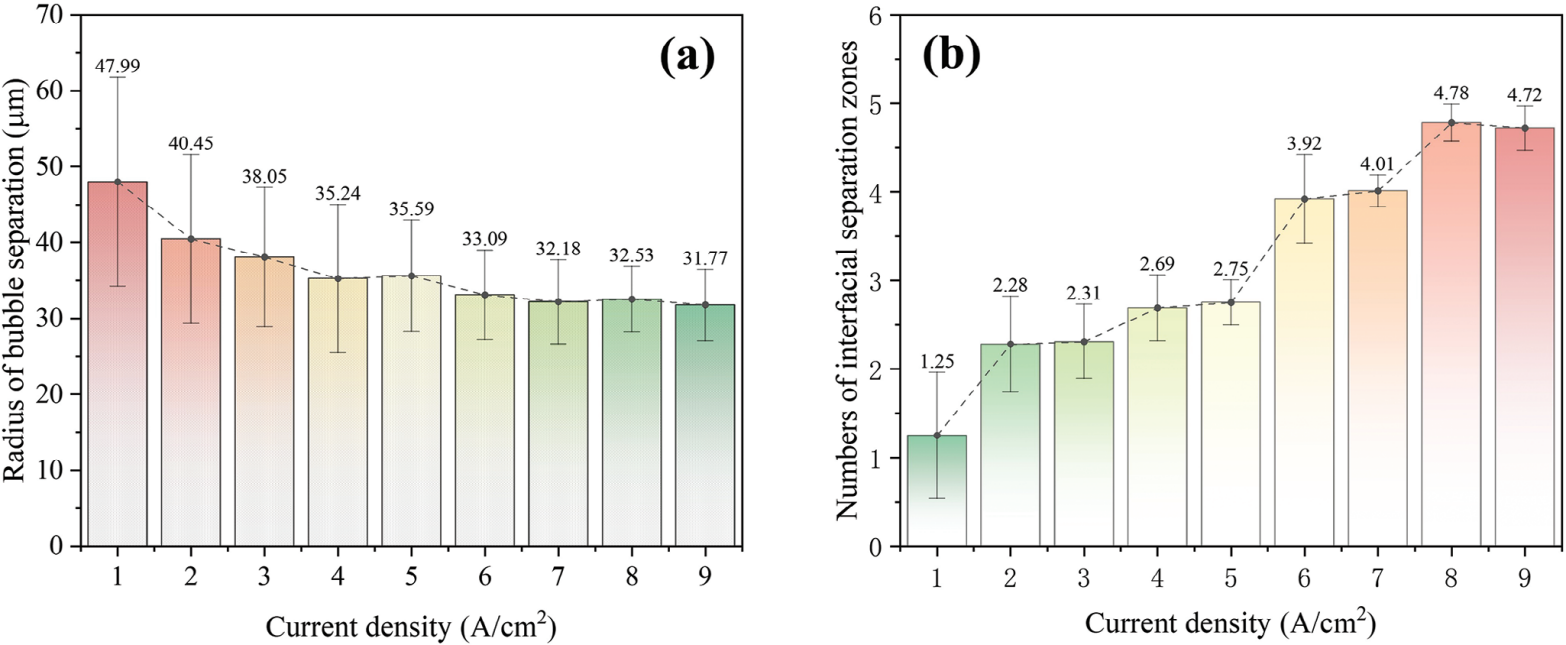
a) Average radius and b) number of ISZs at different current densities observed in each cross‐sectional radiograph.

**Figure** **7** further illustrates the characterization of the ISZ under various current densities. From the perspective of the mean radius, the radius of the ISZ is little affected by the current density, which is consistent with the conclusions of existing studies.^[^ ^13^ ^]^

It is noticeable that the mean radius slowly decreases with the increase of current density and tends to stabilize to 32 µm, and the standard deviation decreases accordingly after the critical current density. It indicates that the bubbles are in a state of rapid detachment and reach relative stability at high current density.

However, from the perspective of the number of ISZs in each radiograph, it is worth noting that the number of ISZs rises in a step‐wise way as the current density increases. In addition, the degree of their number growth is gradually slowing down, especially after exceeding the critical current density, the number of ISZs seems to reach an upper limit, as shown in Section 3.2. Here, the key finding is that the formation of the ISZ is driven by the growth of current density and that its growth is not linearly continuous. Only when a certain current density stage is reached, new ISZs will appear. This new finding can be rationalized by the mechanism of bubble nucleation in porous media, where the essence of bubble formation is determined by the oxygen concentration in the water. Bubbles begin to nucleate only when the local oxygen concentration is much higher than their saturation concentration, ≈350 times^[^ ^35^ ^]^ the saturation concentration, and then the oxygen concentration rapidly drops back to the saturation concentration in the region where the bubbles nucleate, then the concentration gradient drives the bubbles to grow further until they reach a dynamic equilibrium of concentration. Eventually, the bubbles are separated by the drag force of the water flow, which forms the ISZ proposed in this study. What we can learn from this is that when an ISZ is present, it acts as a sink for the dissolved gas and lowers its local concentration.^[^ ^30^ ^]^ In addition, the ISZs are likely to follow a preferential occurrence in some regions with high local oxygen concentrations, as Figure 4 shows. This precisely explains the stepwise growth of the ISZ, only when the current density and gas production reach a higher stage, a new zone of supersaturation is available for the ISZ. Especially when the critical current density is exceeded, it seems difficult to have more ISZs appear in a limited zone.

### 4.2 Oxygen Overpotential Analysis

**Figure 8:**
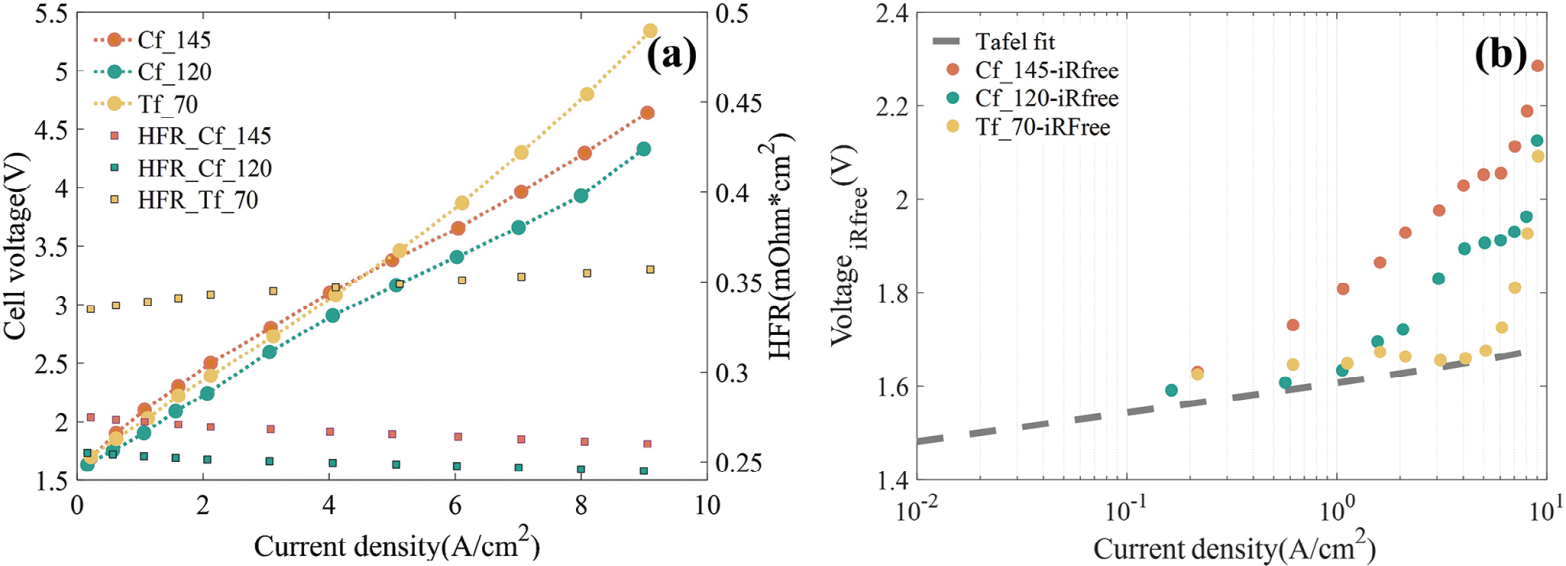
a) In situ polarization curves, and b) the IR‐free cell potentials of three mini‐cells from the polarization stage. (The region sandwiched between the scatter and the tafel fit indicates the overpotential due to oxygen transport.).

Galvanostatic polarization curves are used to compare the electrochemical performance between the three mini‐cells with different PTL, as shown in **Figure** **8**a. None of the three mini‐cells showed excellent polarization performance and no significant mass transfer polarization was observed since they all suffer from severe high‐frequency impedances (HFR) over 250 mΩ × cm^2^. In particular, the HFR of the Tf_70 PTL mini‐cell is higher than carbon‐based Cf_120 and Cf_145, which contributes to the worst polarization performance of the mini‐cell with titanium‐felt at high current density.

To remove the elimination of ohmic overpotentials,^[^ ^36^ ^]^ Figure 8b shows the iR‐free potentials of three mini‐cells. Due to the same CCM, the activation overpotentials of the three mini‐cells should be consistent, as shown by the Tafel fit. Therefore, the difference between the IR‐free potentials and the Tafel‐fit represents the overpotential only caused by oxygen transport. It can be compared that the oxygen overpotentials of mini‐cells with Cf_145, Cf_120, and Tf_70 are 600, 410, and 395 mV respectively. This indicates that the hydrophobically treated Cf_145 may have the poorest oxygen transport capacity while the inherently hydrophilic titanium‐felt has a better oxygen transport capacity.^[^ ^37^, ^38^ ^]^ This is consistent with the hydrophilic design requirements of PTL in the literature,^[^ ^37^, ^39^, ^40^ ^]^ which facilitates oxygen exclusion and water supply.

More importantly, all three mini‐cells showed a trend of a sharp increase in the oxygen overpotential in the exponential coordinates. Starting from 1 A cm^−2^ current density, oxygen overpotentials can be steadily observed. A clear inflection point is observed for all three mini‐cells between 4 and 7 A cm^−2^, which is also the interval of the critical current density described previously. After the critical current density, the oxygen overpotential rises in acceleration, resulting in unstable cell voltage.

Here, the high oxygen overpotential precisely validates the severe oxygen transport behavior at the critical current density. It is well known that the oxygen overpotential is caused by the concentration impedance, which can be further discussed about critical current density. In Section 3.2, it can be recognized that oxygen is exhausted in the form of bubbles and the ISZs will appear when excessive oxygen is produced. Actually, the formation of both bubbles and ISZ reduces the regional local oxygen concentration and establishes a concentration gradient, which helps to relieve the growth of the concentration impedance. However, as approach the critical current density, the growth of the number of ISZs is limited, which makes it difficult to form more zones to facilitate bubble separation for exclusion and to reduce the localized oxygen concentration in the PTL at high current densities. This ultimately leads to an increase in the concentration impedance and oxygen overpotential.

**Figure 9:**
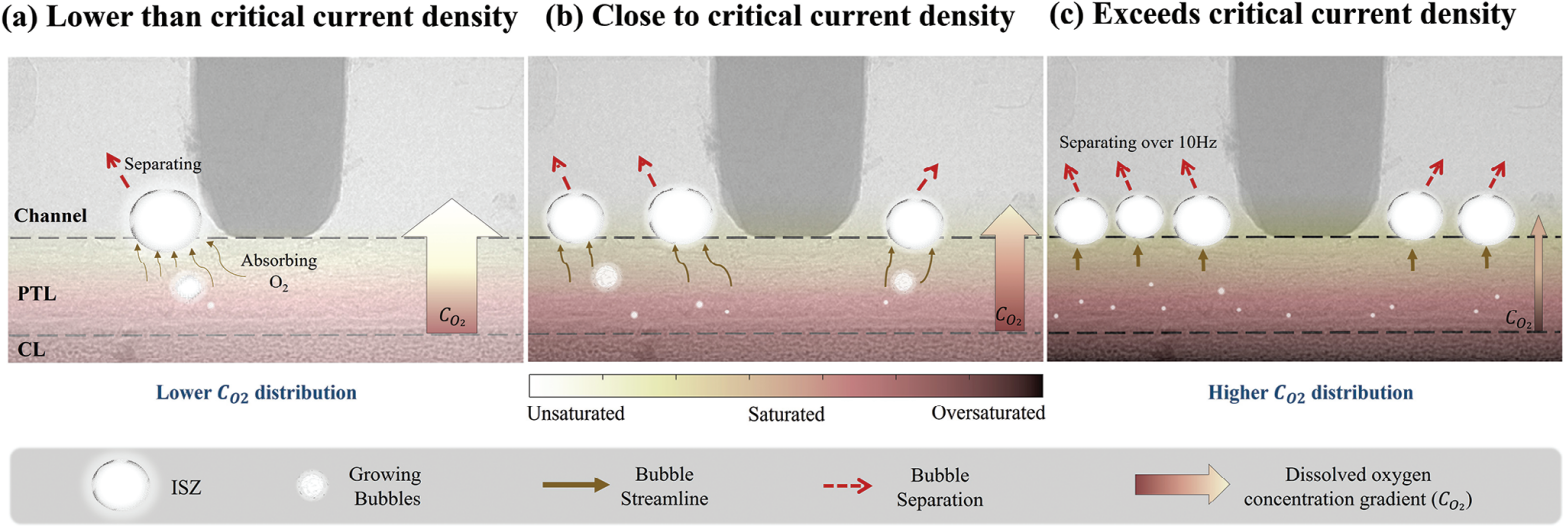
Oxygen transport mechanisms in proton exchange membrane water electrolyzer a) under critical current density, b) at critical current density, and c) exceeds critical current density.

Based on the above analyses of in situ experimental observations and overpotential responses, the PEMWE performance under high current density and the intrinsic oxygen transport mechanisms can be briefly summarized in **Figure** **9**.

Oxygen transport in the electrolyzer still follows the general process of bubble nucleation, growth, and separation. Oxygen tends to nucleate in the region of localized oxygen supersaturation, which is close to the catalytic layer, and the bubble grows further by absorbing the surrounding oxygen until it grows to the ISZ between the flow channel and the PTL, where the bubble begins to detach.

With the increasing current density, the frequency of bubble separation accelerates accordingly, due to more producing oxygen, which leads to a specific phenomenon in the ISZ. When the current density is much less than the critical current density, bubble growth, and intermittent separation can be observed, while few stable ISZs appear. When close to the critical current density, the bubble separation frequency increases further until many ISZs are observed to stabilize, at which point the bubble separation dynamics are difficult to capture. For cell performance, the oxygen overpotential begins to rise. Once the critical density is exceeded, all ISZs seem to stabilize and the image appears to be static, with no new ISZs observed. This may be due to the formation of ISZs being limited by the operating conditions or the space region, so that there are no new interfacial zones to facilitate oxygen exhaustion, resulting in a further blockage of oxygen transfer and ultimately resulting in a high oxygen overpotential.

In this regard, the ideal PTL structure should promote the generation of oxygen in the form of numerous small bubbles at the bottom of the PTL and the formation of more ISZs in the interfacial region to facilitate the rapid separation of the bubbles. Super‐hydrophilic nano‐composite structures for novel PTLs can be proposed.

## 5 Conclusion

The oxygen bubble dynamics behavior inside the PEMWE under high current density was in situ revealed by a novel designed visual mini‐cell coupled with the synchrotron X‐ray beam. Based on the voltage response analysis of mini‐cells with three PTLs, New oxygen evolution and transport mechanisms are proposed for the first time, and detailed results can be summarized as:

1) In situ operation at high current densities revealed that critical current densities (4–7 A cm^−2^) exist for both carbon‐based and titanium‐based PTLs. Once exceeding the critical current density, the cell voltage can no longer be stabilized at a constant current density and the cell exhibits a significant oxygen overpotential of over 395 mV @ 9 A cm^−2^, and hydrophobic treatment leads to even higher oxygen overpotentials.
2) Oxygen evolution and transport in the PEMWE still follow the process of bubble nucleation, growth, and separation within 1 s, Statistically, the radius of the bubble radiuses follows a normal distribution with a mean of 35.3 µm, and the radius of separation tends to be consistent with the increasing current density. While no specific preferential exhaust pathway for oxygen is observed.
3) The stable region where the bubbles begin to separate was observed and the ISZ was proposed for the first time. The ISZ is related to local oxygen concentration and bubble growth processes, and the ISZ preferentially occurs in more hydrophilic regions.
4) The pattern of the ISZ exhibits specific regimes with the critical current density. When below the critical current density, newly appearing zones (ISZs) are constantly observed; Once exceed the critical density, all ISZs seem to stabilize and the image appears to be static, with no new ISZs observed, which results in further blockage of oxygen transfer and a high oxygen overpotential. This is due to the number of ISZs being limited at high current densities, while the ISZ facilitates the absorption of localized oxygen concentrations, promoting oxygen transport and bubble separation.

Ultimately, the mechanism of oxygen transport behavior on cell performance was established. It is identified that the rapid and efficient exhaust of oxygen bubbles is the key to cell performance at high electron density, and the ISZ is found to be an effective pathway for oxygen exhaust. More easily formed ISZs promote faster bubble separation and improve oxygen transfer efficiency, which also provides guidelines for future PTL designs.

## Conflict of Interest

The authors declare no conflict of interest.

## Supporting information

Supporting Information

Supplemental Video

## Data Availability

The data that support the findings of this study are available in the supplementary material of this article.
