# Supplementary material for: Insight into Oxygen Transport in Proton Exchange Membrane Water Electrolyzers by In Situ X‐Ray Characterization
Source: Adv Sci (Weinh). 2024 Sep 26;11(43):2405658. doi: 10.1002/advs.202405658 (PMC11578382; doi:10.1002/advs.202405658)
Supplement: Supplementary file 1 — Supporting Information [file ADVS-11-2405658-s002.docx]

**Supporting Information**

Ping’an Li ^a,*^,Zihan Zhou ^a^, Diankai Qiu ^a, b^, Linfa Peng ^a, b^

^a^ State Key Laboratory of Mechanical System and Vibration, Shanghai Jiao Tong University, Shanghai 200240, P.R. China

^b.^ Shanghai Key Laboratory of Digital Manufacture for Thin-walled Structures, Shanghai Jiao Tong University, Shanghai 200240, P.R. China

(Tel)+86-18818221153

(Fax) +86-21-34204542

E-mail: pinganli@sjtu.edu.cn

# Dynamic results of in situ observations

To better visualise the water and gas transport processes inside the micro cell, oxygen bubbles in the image streams of entire cross-sectional tomographs are marked in red, as shown in videos from Data File_image streams.

The three named videos represent dynamic results of in situ observations under critical current density (1-3A/cm^2^), close to critical current density (4-6A/cm^2^) and over critical current density (7-9A/cm^2^) respectively, as shown in Fig. S1. The video stream contains oxygen transport in the anode on the upper side and hydrogen transport in the cathode on the lower side. All three videos are based on Cf_45 sample capturing, the X-ray energy was selected at 13 keV, coupling 2x lenses and 10x lenses and the temporal sampling rate was 10 Hz. Image stream post-processing was based on image processing toolbox in Matlab2022a and labelling of oxygen bubbles is based on the Labelme package in python3.11.

Ultimately, the videos stream reveal the conclusion as stated in the manuscript. The ISZ is an effective pathway for bubble detachment and is associated with critical current density. Detailed results can be summarized as: With the increasing current density, bubble detachment's frequency accelerates accordingly, leading to a specific phenomenon in the ISZ. When below the critical current density, the bubble detachment frequency is below 10Hz and the ISZ is intermittently observed. When close to the critical current density, the bubble detachment frequency is close to 10Hz until many ISZs are observed to stabilize. Once the critical density is exceeded, all ISZs seem to stabilize and the image appears to be static, and the number of interface separation zones reaches the limit.


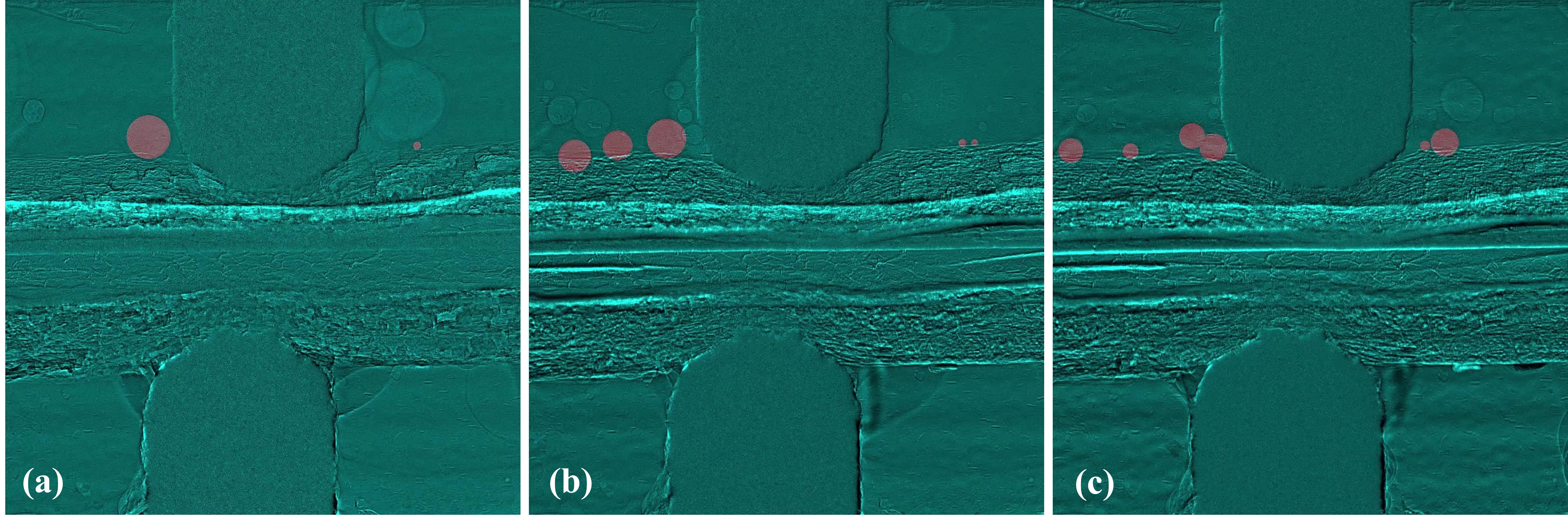


Fig. S1. Dynamic results of in situ observations (a) under critical current density, (b) close to critical current density, (c) over critical current density.
